# A computational relationship between thalamic sensory neural responses and contrast perception

**DOI:** 10.3389/fncir.2015.00054

**Published:** 2015-10-08

**Authors:** Yaoguang Jiang, Gopathy Purushothaman, Vivien A. Casagrande

**Affiliations:** ^1^Department of Psychology, Vanderbilt UniversityNashville, TN, USA; ^2^Department of Cell and Developmental Biology, Vanderbilt UniversityNashville, TN, USA; ^3^Department of Ophthalmology and Visual Sciences, Vanderbilt UniversityNashville, TN, USA

**Keywords:** lateral geniculate nucleus (LGN), perception, contrast, neural model, choice probability

## Abstract

Uncovering the relationship between sensory neural responses and perceptual decisions remains a fundamental problem in neuroscience. Decades of experimental and modeling work in the sensory cortex have demonstrated that a perceptual decision pool is usually composed of tens to hundreds of neurons, the responses of which are significantly correlated not only with each other, but also with the behavioral choices of an animal. Few studies, however, have measured neural activity in the sensory thalamus of awake, behaving animals. Therefore, it remains unclear how many thalamic neurons are recruited and how the information from these neurons is pooled at subsequent cortical stages to form a perceptual decision. In a previous study we measured neural activity in the macaque lateral geniculate nucleus (LGN) during a two alternative forced choice (2AFC) contrast detection task, and found that single LGN neurons were significantly correlated with the monkeys’ behavioral choices, despite their relatively poor contrast sensitivity and a lack of overall interneuronal correlations. We have now computationally tested a number of specific hypotheses relating these measured LGN neural responses to the contrast detection behavior of the animals. We modeled the perceptual decisions with different numbers of neurons and using a variety of pooling/readout strategies, and found that the most successful model consisted of about 50–200 LGN neurons, with individual neurons weighted differentially according to their signal-to-noise ratios (quantified as d-primes). These results supported the hypothesis that in contrast detection the perceptual decision pool consists of multiple thalamic neurons, and that the response fluctuations in these neurons can influence contrast perception, with the more sensitive thalamic neurons likely to exert a greater influence.

## Introduction

From smelling a flower to recognizing the face of a loved one, every perceptual task we face, simple or complex, involves a number of neurons in a wide range of brain areas. Of essential interest to neuroscientists is the number of sensory neurons needed to sustain a perception, and the way these neurons are decoded at a later stage to form various decisions. Theoretically, every perceptual task can be accomplished by engaging only the few sensory neurons that are the most sensitive for that task (i.e., the lower envelope principle; Barlow, 1995; Parker and Newsome, 1998). In reality, however, a variety of factors such as the response variances of single neurons and the positive noise correlations between pairs of neurons constrain the pool size, requiring at least 10–1000 sensory neurons in an average sized decision pool (Shadlen et al., 1996; Cook and Maunsell, 2002; Purushothaman and Bradley, 2005; Cohen and Newsome, 2009; Liu et al., 2013). In sensory cortex, such perceptual decision pools have two prominent features. First, stimulus-independent, random fluctuations of sensory neural responses are known to covary with the perceptual decisions of the animal. The strength of this covariation is quantified as “choice probability” (Britten et al., 1996). Weak but significantly above chance choice probabilities have been observed in a number of sensory cortical areas (Britten et al., 1996; Dodd et al., 2001; Cook and Maunsell, 2002; Grunewald et al., 2002; Uka and Deangelis, 2004; Liu and Newsome, 2005; Purushothaman and Bradley, 2005; Uka et al., 2005; Nienborg and Cumming, 2006; Palmer et al., 2007). Second, cortical sensory neurons are also correlated with each other in their random response fluctuations (Averbeck et al., 2006; Cohen and Kohn, 2011). This correlation, known as the interneuronal noise correlation, is likely to reflect the shared feedforward, feedback, or lateral connections between neurons (Zohary et al., 1994; Shadlen and Newsome, 1998; Bair et al., 2001; Reich et al., 2001; Cohen and Maunsell, 2009). Previous modeling work has revealed that interneuronal correlations can have a profound influence on the choice probability structure of the decision pool (Shadlen et al., 1996; Cohen and Newsome, 2009; Nienborg and Cumming, 2010; Haefner et al., 2013).

Such interneuronal correlation or choice probability measurements, however, are rarely made in subcortical structures (but see Liu et al., 2013). In the mammalian visual system, the retina sends direct input to the lateral geniculate nucleus (LGN) of the thalamus which, in turn, relays this information to the visual cortex. Recently, we reported the first study in which LGN neural responses were examined in detail while the animals were required to make perceptual decisions using the information available within the receptive fields of those LGN neurons (Jiang et al., 2015). In a two alternative forced choice (2AFC) contrast detection task, we found that the majority of single LGN parvocellular (P) and magnocellular (M) neurons were not as sensitive as the monkeys. Importantly, the covariation between neural responses and perceptual decisions, measured as choice probability, was significant for both P and M neurons, even though the average interneuronal correlation between LGN neuron pairs was not different from zero. Additionally, both neural sensitivity and choice probability evolved throughout the stimulus presentation time, with M neurons exhibiting faster and more transient response profiles than P neurons (Jiang et al., 2015).

Taking advantage of this previously characterized dataset and using a computational approach, we investigated in this study how single LGN neurons contribute to our perception of contrast. We built a series of models to explore the interaction between the size of the decision pool, the duration of integration time, and the pooling/readout strategy of the neural system. Because previous experimental and computational work has suggested a positive relationship between neural sensitivity, choice probability, and readout weight (for example see Britten et al., 1996; Shadlen et al., 1996; Purushothaman and Bradley, 2005; Haefner et al., 2013; Liu et al., 2013), we examined not only the standard uniform readout model but also several alternative weighted readout schemes in which individual neurons were assigned different weights based on their sensitivities. We accepted or rejected these models based on their ability to account for the behavioral performance of the monkeys as well as the measured choice probability values for LGN neurons (see Jiang et al., 2015). Aspects of the modeling data presented here have been published in abstract form (Jiang et al., 2012, 2013).

## Materials and Methods

All the experimental procedures regarding surgical preparation, animal training, stimulus presentation, and physiological recordings have been described in detail in previous publications (Jiang et al., 2013, 2015), and are therefore only briefly repeated here when relevant.

### Subjects

Two macaque monkeys (monkey 1: *Macaca radiata*, male, 7 kg, 10 years old; monkey 2: *Macaca mulatta*, male, 8 kg, 12 years old) served as subjects. The monkeys were treated and cared for in accordance with the National Institutes of Health Guide for the Care and Use of Laboratory Animals and the guidelines of Vanderbilt University Animal Care and Use Committee under an approved protocol. The monkeys underwent sterile procedures for the implantation of head posts and recording chambers. The chambers were centered over the right LGN of monkey 1 (AP = 7, ML = 12.5) and the left LGN of monkey 2 (AP = 7, ML = 12).

### Visual Stimulus Presentations and Behavioral Tasks

The monkeys were first trained to fixate on a central fixation spot for an extended period of time. Next, the monkeys were trained to perform a two-alternative forced choice (2AFC) contrast detection task, in which a contrast stimulus was presented either at the receptive field location of the cell being recorded, or at a symmetrical location in the opposite visual hemi-field, for a fixed duration (200 ms). The monkeys saccaded to one of the two target locations to indicate the side on which the stimulus was presented. The stimulus diameter was always the sum of the classical receptive field diameter (center and surround) plus the fixation window diameter. During each recording session, stimuli of 5 or 9 different contrast levels (including 0% contrast, or blank trials, where no physical stimulus was presented) were presented at each location. Different contrast levels and presentation locations (i.e., left or right) were randomly mixed, with equal probabilities of left or right appearance and higher proportions of low to medium contrast trials to ensure accurate estimations of the psychophysical threshold.

### Psychometric Functions

The proportion of correct responses from the monkeys was plotted for each contrast, and a Weibull function was fitted to the data:

(1) $$P(c)=1-0.5{\text{ * e}}^{-{\left(\frac{c}{\alpha }\right)}^{\beta }}$$

Where *P(c)* is the probability of correct responses at contrast level *c*, *α* is the contrast level that supports threshold performance (82% correct), and *β* is the slope of the function.

### Cell Mapping and Classification

LGN cells were hand mapped using first a flashlight and then an elongated bar with a sharp contrast profile. Cells were classified as ON-center or OFF-center cells, and Parvocellular (P) or Magnocellular (M) cells, based on their visually driven responses (Norton and Casagrande, 1982; Norton et al., 1988; Xu et al., 2001; Royal et al., 2006; Jiang et al., 2015).

### Neurometric Functions

Basic procedures in computing neurometric functions were similar to those described in previous studies (Barlow et al., 1971; Britten et al., 1992; Purushothaman and Bradley, 2005). For every contrast level, an ROC (Receiver Operating Characteristic) curve was computed (Green and Swets, 1966). Each ROC curve plotted, for all possible signal detection criteria (spikes), the proportion of stimulus-inside-receptive-field trials where the spike count exceeded a certain criterion, against the proportion of stimulus-outside-receptive-field trials that exceeded the same criterion. Next each area-under-ROC curve value was calculated and plotted against its corresponding contrast, a Weibull function ([1], as described above) was fitted, and the neurometric threshold and slope were obtained from the fitted curve. All the LGN cells that could be clearly mapped and maintained long enough to characterize both the psychophysical and neural responses (>150 trials, overall psychophysical performance >65% correct) in the detection task were included in this analysis (overall: *n* = 89 neurons; monkey 1: *n* = 61; monkey 2: *n* = 28). We identified in this dataset 41 ON-center P neurons, 27 OFF-center P neurons, 19 ON-center M neurons, and 2 OFF-center M neurons. We found that the average neurometric threshold (54.4 ± 4.78% contrast, *n* = 89 neurons, in 0–150 ms integration time windows) was significantly different from the simultaneously measured psychometric threshold (5.76 ± 0.72% contrast, *P* = 0.000, Wilcoxon signed rank test). The average ratio of neurometric to psychometric threshold was 40.74 ± 10.75, indicating that the average LGN neuron was much less sensitive than the monkey in contrast detection (Jiang et al., 2015).

### Choice Probability

Basic procedures in computing choice probabilities also were similar to those described in previous studies (Britten et al., 1996; Purushothaman and Bradley, 2005). The choice probability for a certain contrast was measured by plotting, as an ROC curve, the proportion of choice-inside-receptive-field trials (i.e., trials in which the monkey saccaded towards the receptive field location) against the proportion of choice-outside-receptive-field trials that exceeded the spike count criteria, and computing the area under that curve. The significance of individual or population choice probabilities was assessed using permutation tests (Britten et al., 1996; Jiang et al., 2015). To accurately estimate choice probability, only neural recordings that met the following criteria were included in this analysis: (1) Behavior *ratio* (choice-inside/choice-outside-receptive-field trials) > 0.25 and <4; and (2) For every contrast level that was included in the choice probability computation, at least 10 choice-inside and 10 choice-outside-receptive-field trials were recorded. Out of the 89 neurons in the above dataset, 75 (54 P neurons, 21 M neurons) were included in the choice probability analysis according to these criteria. We found in this dataset that, in the absence of any physical stimulus (i.e., 0% contrast, blank trials only), the average choice probability was 0.54 ± 0.01 for LGN P neurons and 0.54 ± 0.01 for LGN M neurons, both above chance according to permutation tests (P neuron: *P* = 0.015, M neuron: *P* = 0.033; Jiang et al., 2015).

### Pooling Model

The basic structure of our pooling model was similar to other bottom up pooling models previously proposed to account for the psychophysical threshold and choice probabilities measured during behavioral tasks (Shadlen et al., 1996; Purushothaman and Bradley, 2005; Cohen and Newsome, 2009; Haefner et al., 2013; Liu et al., 2013). Briefly, to simulate a perceptual decision pool of n units, n single neurons were randomly chosen, with replacement, from our entire dataset. To construct a single trial at a given contrast, we simulated each neuron’s response by randomly drawing a number from a Gaussian distribution; the mean and variance of this distribution were determined by that neuron’s measured response at that contrast level. In each trial, the model made a “choice” by comparing the summed activity of the neural pool at the test contrast level to the summed activity of the same neurons at the reference contrast (i.e., blank, 0% contrast trials). This procedure was repeated 50 times (i.e., to simulate 50 trials) for each of the 5 contrast levels, and the simulated “psychophysical” performance was recorded as the percentage of correct “choices” at each contrast. This performance was fitted with a cumulative Weibull function [1], and threshold and slope parameters were extracted as described above. The choice probability for each simulated neuron was quantified as the covariation between the simulated neural response and the simulated “psychophysical choice” at 0% contrast. The parameters for the model included the number of neurons in the pool (*n* = 1–512 neurons), the integration time window (*t* = 25–200 ms), the Fano factor (*f* = 0.25–3.0), the interneuronal noise correlation (*r* = 0–0.3), and the downstream pooling noise (*p* = 0–4.0). For each parameter combination, the set of simulations described above (50 trials * 5 contrast levels) was repeated 200 times, each time with a new random sample of n neurons (with replacement), thus giving reliable estimations of the model performance. The overall fitness of the model was evaluated by computing a Goodness-of-Fit (GoF) index:

(2) $$\begin{array}{l} GoF=(1-1/3*(|simulated\;threshold-measured\\ threshold|/measured\;threshold+|simulated\;P\;choice\\ probability-measured\;P\;choice\;probability|/measured\\ P\;choice\;probability+|simulated\;M\;choice\;probability\\ -\;measured\;M\;choice\;probability|/measured\;M\;choice\\ probability))*100\% \end{array}$$

### Model Parameters

**Figure 1:**
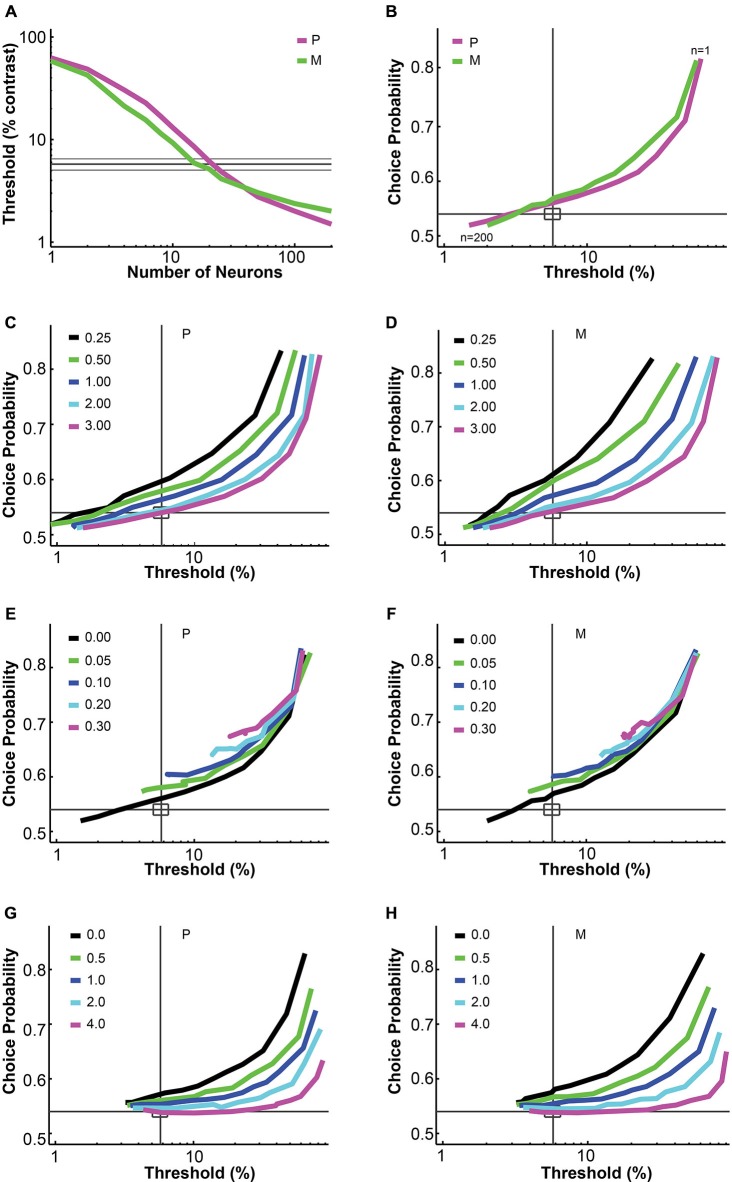
**Parametric analysis of the pooling model. (A,B)** Increasing the number of neurons in the pool decreased the simulated threshold and choice probability. Interneuronal correlation and pooling noise were assumed to be 0; integration time was 0–150 ms. Magenta: simulated P neuron; green: simulated M neuron; thick gray line: measured mean psychophysical threshold/choice probability; thin gray line/rectangle: mean ± SEM. **(C,D)** Increasing the Fano factor increased simulated threshold but maintained choice probability, for both P neurons **(C)** and M neurons **(D)**. Interneuronal correlation and pooling noise were assumed to be 0; integration time was 0–150 ms. As in **(B)**, each simulation line moved from the upper right corner to the lower left corner as more neurons were added in the pool. Gray line: measured mean psychophysical threshold/choice probability; rectangle: mean ± SEM. **(E,F)** Increasing interneuronal correlation increased simulated threshold and choice probability, for both P neurons **(E)** and M neurons **(F)**. The Fano factor was assumed to be 1.03 (measured value); pooling noise was assumed to be 0; integration time was 0–150 ms. As in **(B)**, each simulation line moved from the upper right corner to the lower left corner as more neurons were added in the pool. Legends as in **(C,D)**. **(G,H)** Increasing pooling noise increased simulated threshold and decreased choice probability, for both P neurons **(G)** and M neurons **(H)**. The Fano factor was assumed to be 1.03 (measured value); interneuronal correlation was assumed to be 0.028 (measured value); integration time was 0–150 ms. As in **(B)**, each simulation line moved from the upper right corner to the lower left corner as more neurons were added in the pool. Legends as in **(C,D)**. **(A,B)** and **(E–H)** were adapted from Jiang et al. (2015).

In our simulations we typically used fixed or experimentally measured values (Jiang et al., 2015) for variables such as the Fano factor, the interneural correlation, and the pooling noise (with the exception of Figure 1, where these parameters were systematically varied to probe the basic properties of the pooling model). We consider our choices for these parameter values to be neurobiologically realistic and meaningful for the following reasons: (1) The Fano factor: Recordings in anesthetized as well as alert animals have reported significant variabilities in the responses of single cortical neurons, with the Fano factor (response variance/mean) averaging 1.0–3.0 (Tolhurst et al., 1983; McAdams and Maunsell, 1999; Oram et al., 1999; Gu et al., 2007). The Fano factor of subcortical visual neurons, however, is relatively low (i.e., <1.0). This is true for retinal ganglion cells (Levine et al., 1992; Berry et al., 1997; Reich et al., 1997) as well as LGN cells (Kara et al., 2000). The Fano factors measured in our detection task (Jiang et al., 2015) and used in our models (0.8–1.4, depending on integration time) were in agreement these previously reported measurements. (2) Interneuronal correlation: In sensory cortex, interneuronal correlations between pairs of nearby neurons are typically weak but positive (~0.1–0.2; Averbeck et al., 2006). For the LGN P-P and M-M neuron pairs, because convergent feedforward, divergent feedforward, and lateral connections are sparser than those in the cortex (Casagrande and Xu, 2004; Nassi and Callaway, 2009), it is not surprising that we found an average interneuronal correlation (0.028) that was not significantly different from 0.0. The interneuronal correlation for a P-M neuron pair is very likely to be even smaller, as the P and M pathways receive different retinal inputs, remain segregated in different layers of the LGN (Casagrande and Norton, 1991; Nassi and Callaway, 2009), and retain separate feedback loops with V1 (Ichida and Casagrande, 2002; Briggs and Usrey, 2009, 2011; Ichida et al., 2014). In our model the P-M correlation was fixed at 0.01, but our simulations could always approach >99% GoF at some parameter combinations, given any P-M correlation values between 0.0–0.05 (data not shown). (3) Pooling noise: The downstream pooling noise can be thought of as the average Fano factor of the cortical neurons onto which LGN neurons converge (Shadlen et al., 1996). In our simulations this number was fixed at 2.0, which is the average estimation of the Fano factor in cortex (see above). The success of our simulations (i.e., approaching >99% GoF at some parameter combinations), however, did not depend on this assumption. Similar model results could be obtained by simply assuming a true Poisson distribution for all downstream neurons (i.e., pooling noise = 1.0).

### Uniform Pooling and Alternative Pooling Strategies

In this paper, we examined uniform pooling models as well as several alternative weighted pooling models. All of these models shared the same overall structure:

(3) $$X_{\text{pooled}}=\sum_{i\;=\;1}^{n}w_{i}x_{i}$$

Where X_pooled_ is the summed activity of the perceptual decision pool, *x_i_* is the response of a single neuron, and *w_i_* is the readout weight assigned to this neuron. Within this structure, the uniform pooling model simply assigned equal weights (i.e., *w_i_* = 1.0) to all neurons in the decision pool, regardless of their sensitivities (Shadlen et al., 1996). An alternative weighted pooling strategy, in contrast, calculated the sensitivity of each individual neuron and assigned weights accordingly. Depending on how this neural sensitivity was quantified, there were three main categories of weighted pooling schemes: (1) Amplitude-per-trial (amp/trial) weighted scheme, where every neuron was weighted according to its response amplitude in every trial (*w*_i_ ∝ *x_i_*), with the neuron with the highest spike rate carrying a weight of 1.0; (2) Mean amplitude (mean amp) weighted scheme, where every neuron was weighted according to its average response amplitude $\left(w_{i}\propto \bar{x_{i}}\right)$ at high contrast (80–99%), with the neuron with the highest average spike rate carrying a weight of 1.0; and (3) D-prime weighted scheme, where every neuron was weighted by its d-prime value $\left(w_{i}\propto d_{i}^{\prime }\right)$ at high contrast (80–99%), with the neuron with the greatest d-prime carrying a weight of 1.0. Here d-prime was defined as:

(4) $$d_{{}_{i}}^{\prime }=\left({\bar{x}}_{i}-{\bar{x0}}_{i}\right)/\sqrt{\frac{s_{i}^{2}+s0_{i}^{2}}{2}}$$

Where $\bar{x_{i}}$ is the neuron’s mean response amplitude at high contrast (80–99%), $\bar{x0_{i}}$ is its mean response amplitude at reference contrast (i.e., blank, 0% contrast), and *s_i_* and *s0_i_* represent the corresponding standard deviations. Because of their potential deviations from normality, for both weight and d-prime distributions we reported medians as well as means. Additionally, to characterize the spread of a distribution, we reported the interquartile range:

(5) $$Interquatile\;Range\;(IQR)=Q_{3}-Q_{1}$$

Where *Q_1_* is the 1st quartile (i.e., 25% percentile), and *Q_3_* is the 3rd quartile (i.e., 75% percentile) in the range. To characterize the skewness of a distribution, we reported the skewness index:

(6) $$Skewness\;index\;(SI)=\frac{\frac{1}{n}{\sum_{i=1}^{n}\left(x_{i}-\bar{x}\right)}^{3}}{{\left(\sqrt{\frac{1}{n}\sum_{i=1}^{n}{\left(x_{i}-\bar{x}\right)}^{2}}\right)}^{3}}$$

Where *n* is the sample size, and $\bar{x}$ is the mean of the sample distribution.

## Results

As previously reported (Jiang et al., 2015), we found that single LGN P and M neurons, although not as sensitive as the monkeys in detecting contrast, were significantly correlated with the behavioral choices of the monkeys during a 2AFC contrast detection task. Based on these experimental data, we report in this paper a series of modeling results in the following order: first we describe the basic parameters of the uniform pooling model and its performance in different time frames, then we compare several alternative weighted pooling schemes to the uniform pooling model, and finally we zoom in on one of the best performing weighted schemes and examine its structure in detail.

### Uniform Pooling Model: Parameters

The uniform pooling model we built was similar to a number of previous models used to account for psychophysical performance and choice probability measurements based on sensory neural responses (Shadlen et al., 1996; Purushothaman and Bradley, 2005; Cohen and Newsome, 2009; Haefner et al., 2013; Liu et al., 2013). Inputs to the model were single LGN P and M neural responses at different contrasts. Outputs were the simulated “psychophysical” threshold and the simulated choice probabilities for individual neurons. In agreement with Shadlen et al. (1996), this uniform pooling model behaved predictably when certain model parameters were changed. Specifically, increasing the number of neurons in the pool (n) decreased the psychophysical threshold and choice probability values (Figures 1A,B). Increasing the Fano factor increased the simulated threshold but maintained the same choice probability values (Figures 1C,D). Increasing the interneuronal correlation increased the threshold as well as choice probability values (Figures 1E,F). Increasing the downstream pooling noise increased the threshold and decreased choice probability values (Figures 1G,H). For the simulation results reported in the following sections, the Fano factor and interneuronal noise correlation were fixed at experimentally measured values and the pooling noise, which could be considered as the Fano factor of downstream neurons onto which LGN neurons converge, was assumed to be 2.0 (see “Material and Methods” Section).

### Uniform Pooling Model: Performance

**Figure 2:**
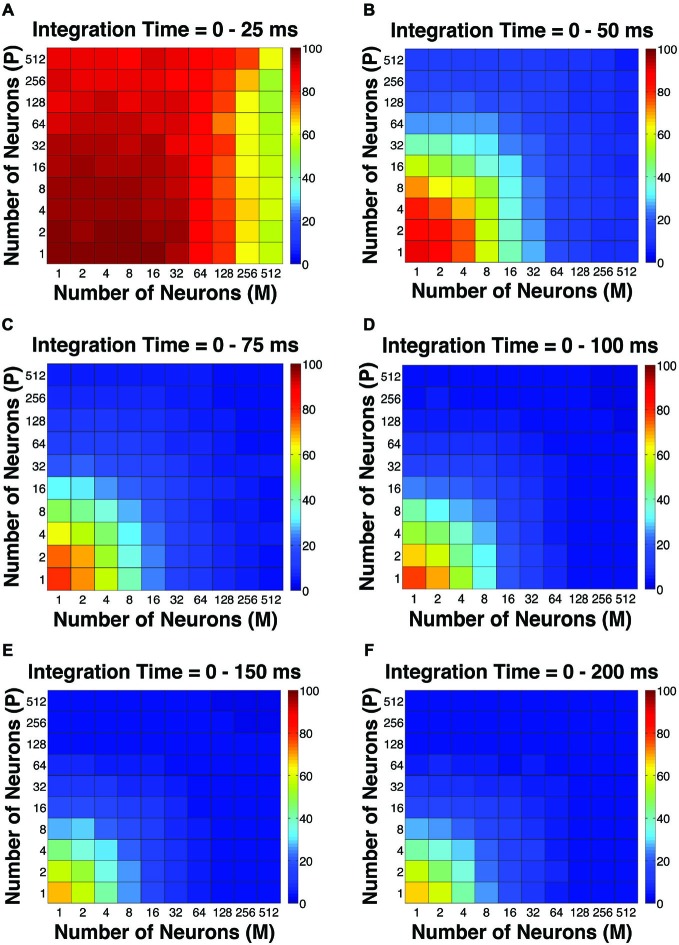
**The simulated “psychophysical” thresholds for different pool sizes (*n*) and integration time windows (*t*)**. Red indicates high threshold (100% contrast), whereas blue indicates low threshold (0% contrast). **(A)** *t* = 0–25 ms. **(B)** *t* = 0–50 ms. **(C)** *t* = 0–75 ms. **(D)** *t* = 0–100 ms. **(E)** *t* = 0–150 ms. **(F)** *t* = 0–200 ms.

**Figure 3:**
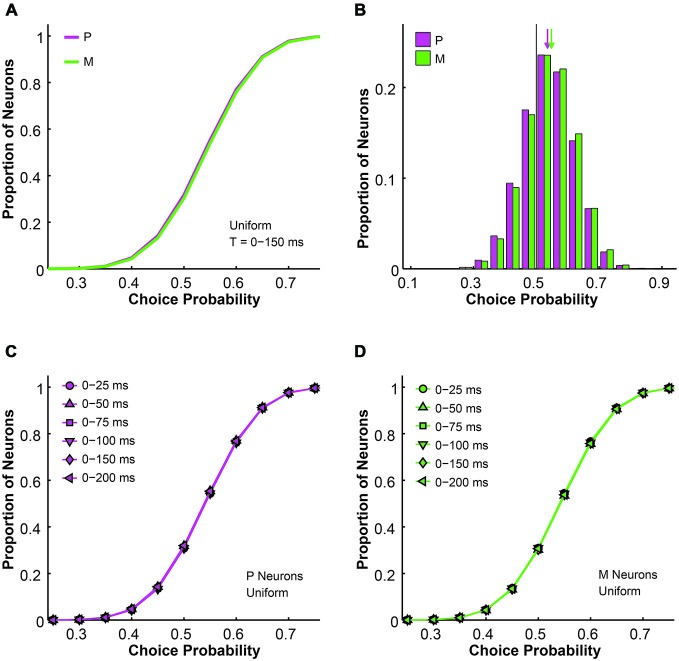
**The simulated choice probabilities of P and M neurons for different pool sizes (*n*) and integration time windows (*t*)**. **(A)** Cumulative choice probability distributions for P (magenta) and M (green) neurons in 200 simulations (*n* = 512 neurons, *t* = 0–150 ms). **(B)** Choice probability distributions for P (magenta) and M (green) neurons in 200 simulations (*n* = 512 neurons, *t* = 0–150 ms). Arrow: mean choice probability; solid line: choice probability = 0.5. **(C)** Cumulative choice probability distributions for P (magenta) neurons in different integration time windows (*n* = 512 neurons, 200 simulations for each integration time). **(D)** Cumulative choice probability distributions for M (green) neurons in different integration time windows (*n* = 512 neurons, 200 simulations for each integration time).

The first pooling scheme we investigated was the uniform pooling model, in which the responses of all neurons were weighted equally and summed up to form perceptual decisions. In this model, the simulated psychometric threshold consistently decreased as: (1) more neurons (n) were added into the pool; and (2) the integration time window (t) was extended. Specifically, we found that: (1) at extremely short intervals (25 ms), incorporating a large number of neurons from both the P and M populations (*n* = 512 P neurons, 512 M neurons) still failed to achieve great psychophysical sensitivities (i.e., threshold <10% contrast), but incorporating a large number of M neurons (*n* = 256–512) rather than P neurons was more beneficial to model performance (Figure 2A); (2) at relatively brief intervals (50 ms), preferably incorporating a large number of M neurons (*n* = 256–512) rather than P neurons produced good psychophysical performance (i.e., threshold <10% contrast; Figure 2B); (3) at medium to long intervals (75–200 ms), a wider range of M/P neuron combinations (*n* = 64–512) yielded good model performance (Figures 2C–F).

Next we analyzed the simulated choice probabilities for the P and M populations and compared them to the measured choice probability distributions (Jiang et al., 2015). Briefly, in the 2AFC contrast detection task, we found that in the absence of any physical stimulus (i.e., 0% contrast, blank trials only), the average choice probability was 0.54 ± 0.01 for LGN P neurons and 0.54 ± 0.01 for LGN M neurons, both above chance according to permutation tests (P neuron: *P* = 0.015, M neuron: *P* = 0.033). In the uniform pooling model, the simulated choice probability distributions for P and M neurons (*n* = 512 neurons, *t* = 0–150 ms, P choice probability = 0.54 ± 0.00, M choice probability = 0.54 ± 0.00) resembled their experimentally measured counterparts (*P* > 0.05, permutation tests; Figures 3A,B). These choice probability patterns remained unchanged throughout the 200 ms stimulus presentation time (*n* = 512 neurons, *P* > 0.05, 1-way ANOVAs; Figures 3C,D).

**Figure 4:**
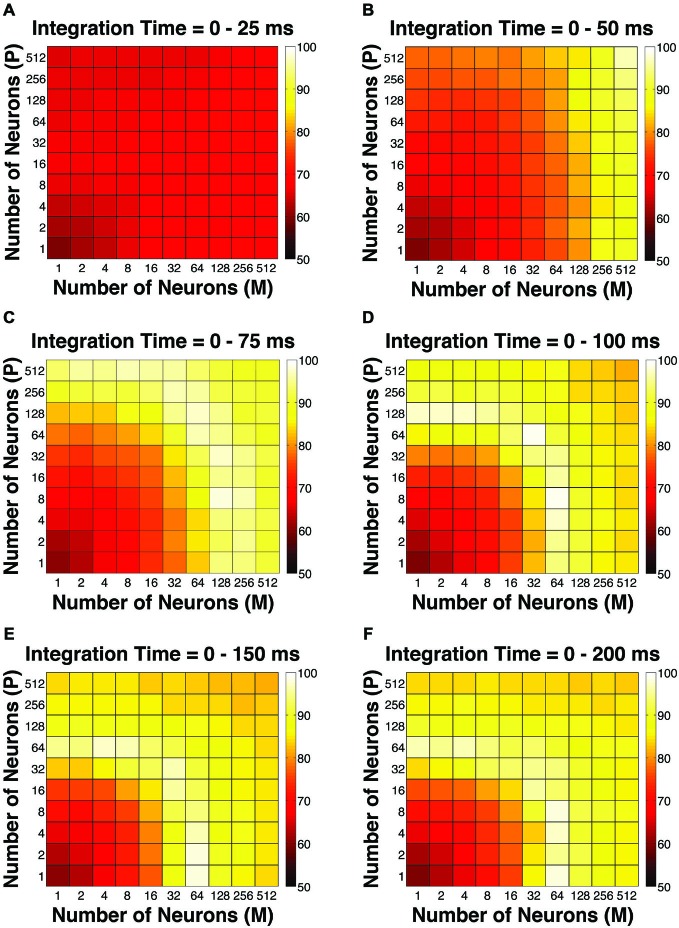
**The Goodness-of-Fit (GoF) indices for different pool sizes (*n*) and integration time windows (*t*)**. A GoF of 100% (white) indicates that the model perfectly matches the observed psychometric threshold as well as choice probabilities for both P and M neurons. **(A)** *t* = 0–25 ms. **(B)** *t* = 0–50 ms. **(C)** *t* = 0–75 ms. **(D)** *t* = 0–100 ms. **(E)** *t* = 0–150 ms. **(F)** *t* = 0–200 ms. **(A,B)** and **(D–F)** were adapted from Jiang et al. (2015).

To evaluate the overall performance of this model, a Goodness-of-Fit (GoF) index (see equation [2]) was reported for each (n, t) parameter combination. A GoF (ranging from 0–100%) reflected three factors equally: (1) how close the simulated “psychophysical” threshold approached the measured psychophysical threshold; (2) how close the simulated P population choice probability approached the measured average choice probability for P neurons; and (3) how close the simulated M population choice probability approached the measured average choice probability for M neurons. A GoF of 100% indicated that our simulation perfectly matched the observed psychometric threshold and the choice probabilities for both types of neurons. By changing the duration of the integration window (25–200 ms), we found that: (1) at extremely short intervals (25 ms), even incorporating a large number of neurons from both groups (*n* = 512 P neurons, 512 M neurons) still failed to reproduce the observed threshold and choice probabilities (Figure 4A); (2) in 50 ms, preferably incorporating a large number of M neurons (*n* = 256–512) could explain the observed threshold and choice probabilities (Figure 4B); (3) in 75 ms, incorporating a large number of either P or M neurons (*n* = 128–512) could achieve good overall model performance (Figure 4C); and (4) at medium to long intervals (100–200 ms), a smaller number of P and M neurons were needed (*n* = 32–128) to achieve good model performance, but further increasing the number of neurons resulted in a decrease in model performance (Figures 4D–F).

### Alternative Pooling Schemes

**Figure 5:**
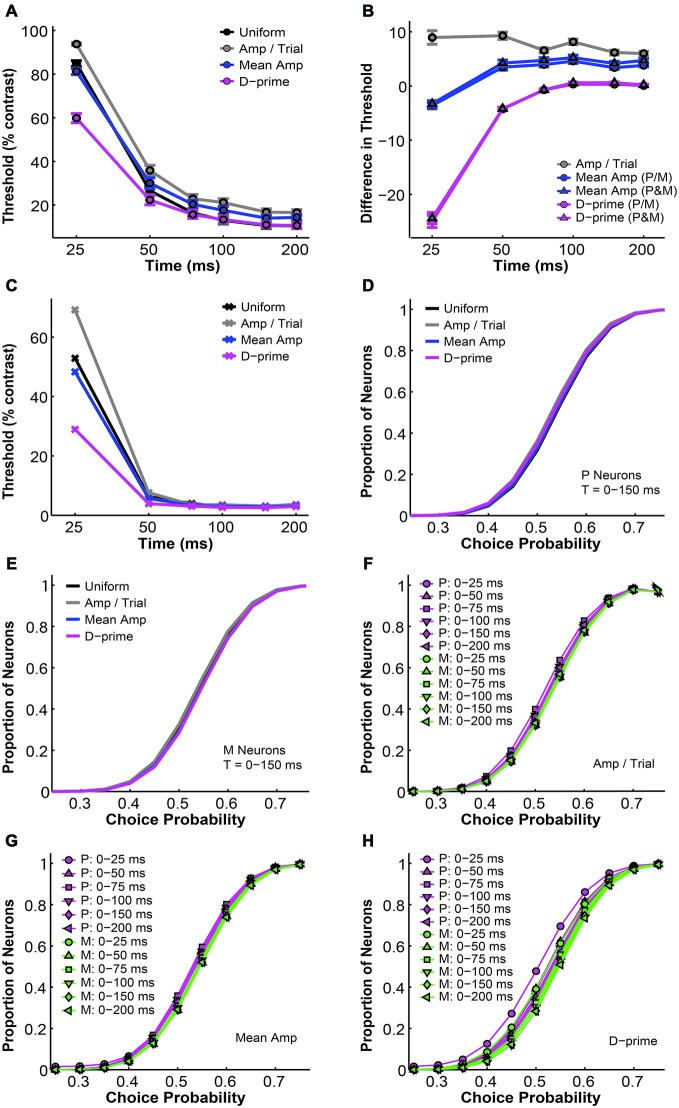
**A comparison of threshold and choice probability values derived from different pooling schemes. (A)** The average psychometric threshold decreased with time in all pooling schemes (*n* = 1–512 neurons). Black: uniform pooling (as above); gray: every neuron weighted by its response amplitude in every trial; blue: every neuron weighted by its average response amplitude at high contrast (80–99%); magenta: every neuron weighted by its d-prime value at high contrast (80–99%); error bar: mean ± SEM. **(B)** Different pooling schemes yielded significantly different psychometric thresholds when compared with the uniform pooling model (*n* = 1–512 neurons). Gray: every neuron weighted by its response amplitude in every trial; blue with circle: every neuron weighted by its average response amplitude at high contrast (80–99%), with P and M neurons weighted separately in reference to their respective maximal responses; blue with triangle: every neuron weighted by its average response amplitude at high contrast (80–99%), with P and M neurons weighted together in reference to one maximal response; magenta with circle: every neuron weighted by its d-prime value at high contrast (80–99%), with P and M neurons weighted separately in reference to their respective maximal d-primes; magenta with triangle: every neuron weighted by its d-prime value at high contrast (80–99%), with P and M neurons weighted together in reference to one maximal d-prime; *y* axis: the difference in psychometric threshold (alternative pooling scheme—uniform, % contrast); error bar: mean ± SEM. **(C)** The minimal psychometric threshold achieved by the model decreased with time in all pooling schemes. **(D,E)** Cumulative choice probability distributions for P **(D)** and M **(E)** neurons (*n* = 512 neurons, *t* = 0–150 ms) in different pooling schemes. **(F–H)** Cumulative choice probability distributions for P (magenta) and M (green) neurons in different integration time windows (*n* = 512 neurons), with every neuron weighted by its response amplitude in every trial **(F)**, by its average response amplitude **(G)**, or by its d-prime **(H)**.

In this section we examine several alternative pooling schemes where, instead of assigning the same weight to all neurons, each individual neuron was weighted differentially based on its response rate or sensitivity. We investigated three main categories of weighted pooling schemes, namely the amplitude-per-trial (amp/trial) weighted, the mean amplitude (mean amp) weighted, and the d-prime weighted schemes (see “Material and Methods” Section). First we compared the simulated psychometric thresholds and found that for all pooling schemes the average psychometric threshold decreased with time (*n* = 1–512 neurons, *F* = 920.22, *P* = 0.00, 2-way ANOVA main effect for time). Furthermore, there was a significant difference in psychometric thresholds among different pooling schemes (*F* = 54.82, *P* = 0.00, 2-way ANOVA main effect for pooling strategy), and this difference changed across time (*F* = 8.43, *P* = 0.00, 2-way ANOVA interaction effect; Figure 5A). Next we examined whether these alternative pooling strategies improved the sensitivity of the model when compared to the uniform pooling strategy. Here the mean amplitude weighted and the d-prime weighted models could be further divided into two subcategories, respectively, depending on whether P and M neurons were weighted separately or together in the model. Among all of these alternative pooling schemes, we found that only the d-prime weighted schemes consistently improved the psychophysical performance when compared with the uniform pooling scheme (mean difference, d-prime 1 = −4.88 ± 0.42% contrast; mean difference, d-prime 2 = −4.67 ± 0.43% contrast; *P* < 0.05, Tukey’s HSD tests for multiple comparisons). The mean amplitude weighted schemes and the amplitude per trial weighted scheme all failed to perform as well as the uniform pooling scheme in terms of the threshold (mean difference, mean amp 1 = 2.59 ± 0.21% contrast; mean difference, mean amp 2 = 3.31 ± 0.23% contrast; mean difference, amp/trial = 7.51 ± 0.28% contrast; *P* < 0.05, Tukey’s HSD tests for multiple comparisons). Additionally, the two subtypes of mean amplitude weighted models did not differ from each other in terms of their simulated thresholds, and the two subtypes of d-prime weighted models did not differ from each other either (*P* > 0.05, Tukey’s HSD tests for multiple comparisons; Figure 5B). Finally, the minimal psychophysical threshold achieved by the model also decreased with time in all pooling schemes and plateaued at around 50–75 ms after stimulus onset (*n* = 1–512 neurons, minimal threshold = 2–3% contrast; Figure 5C). Taken together, Figures 5A–C demonstrated that the d-prime weighted pooling strategies were the most optimal in terms of the simulated psychophysical performance, and this advantage over other pooling strategies was the most apparent in short integration time windows (25–50 ms).

Next, we compared the simulated choice probabilities in these different pooling schemes. First, in a fixed time window (*t* = 0–150 ms), the overall choice probability distributions for simulated P or M neurons did not differ significantly among the pooling schemes (*n* = 512 neurons, *P* > 0.05, 1-way ANOVAs; Figures 5D,E). Second, these choice probability distributions did not change with time in the case of the amplitude per trial and mean amplitude weighted models (*n* = 512 neurons, *P* > 0.05, 2-way ANOVAs main effect for time; Figures 5F,G). In the d-prime weighted pooling model, however, the choice probability distributions did shift significantly as the integration time window was extended (*n* = 512 neurons, *F* = 4.86, *P* = 0.00, 2-way ANOVA main effect for time; Figure 5H), corresponding well to the temporal dynamics of choice probability that were experimentally measured in LGN P and M neurons (see Figure 7 of Jiang et al., 2015).

**Figure 6:**
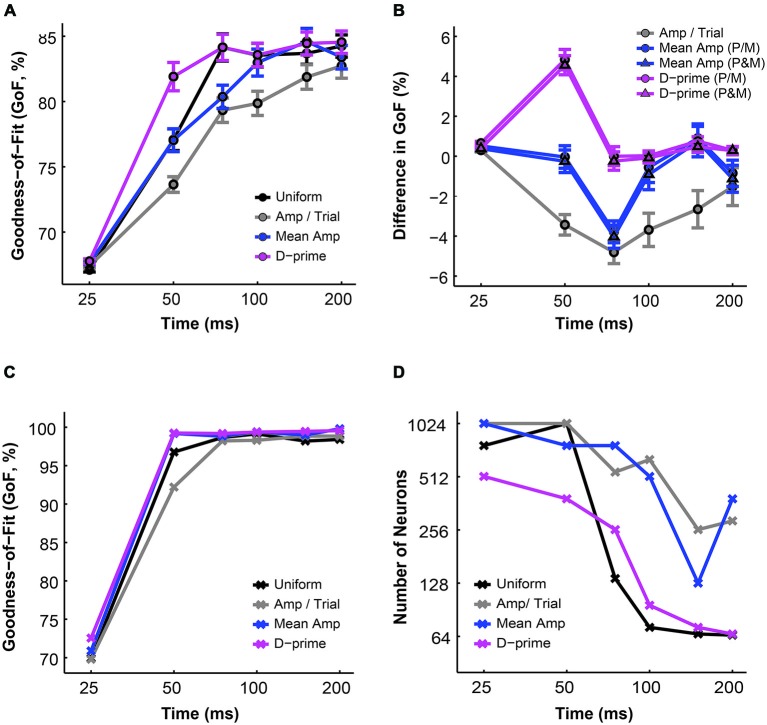
**A comparison of the GoF indices derived from different pooling schemes. (A)** The average GoF increased with time in all pooling schemes (*n* = 1–512 neurons). Black: uniform pooling (as above); gray: every neuron weighted by its response amplitude in every trial; blue: every neuron weighted by its average response amplitude at high contrast (80–99%); magenta: every neuron weighted by its d-prime value at high contrast (80–99%); error bar: mean ± SEM. **(B)** Different pooling schemes yielded significantly different GoFs when compared with the uniform pooling model (*n* = 1–512 neurons). Gray: every neuron weighted by its response amplitude in every trial; blue with circle: every neuron weighted by its average response amplitude at high contrast (80–99%), with P and M neurons weighted separately in reference to their respective maximal responses; blue with triangle: every neuron weighted by its average response amplitude at high contrast (80–99%), with P and M neurons weighted together in reference to one maximal response; magenta with circle: every neuron weighted by its d-prime value at high contrast (80–99%), with P and M neurons weighted separately in reference to their respective maximal d-primes; magenta with triangle: every neuron weighted by its d-prime value at high contrast (80–99%), with P and M neurons weighted together in reference to one maximal d-prime; y axis: the difference in GoF (alternative pooling scheme—uniform, % GoF); error bar: mean ± SEM.** (C)** The maximal GoF achieved by the model increased with time in all pooling schemes. **(D)** The number of neurons needed to achieve the maximal GoF decreased with time in all pooling schemes.

Finally, we investigated the overall performance of different pooling strategies by comparing their GoF indices in different time windows. We found that for all pooling schemes the overall model performance improved with time (*n* = 1–512 neurons, *F* = 223.78, *P* = 0.00, 2-way ANOVA main effect for time). Furthermore, there was a significant difference in overall fitness among different pooling schemes (*F* = 19.06, *P* = 0.00, 2-way ANOVA main effect for pooling strategy), and this difference changed across time (*F* = 2.75, *P* = 0.00, 2-way ANOVA interaction effect; Figure 6A). Next, we examined whether the alternative pooling strategies improved upon the performance of the uniform pooling model. We found that, again, only the d-prime weighted schemes consistently improved the overall fitness of the model (mean difference, d-prime *1* = 1.10 ± 0.15% GoF; mean difference, d-prime 2 = 0.95 ± 0.15% GoF; *P* < 0.05, Tukey’s HSD tests for multiple comparisons). The mean amplitude weighted schemes did not differ significantly from the uniform pooling scheme (mean difference, mean/amp 1 = −0.64 ± 0.25% GoF; mean difference, mean amp 2 = −0.79 ± 0.26% GoF; *P* > 0.05, Tukey’s HSD tests for multiple comparisons), whereas the amplitude per trial weighted scheme failed to perform as well as the uniform pooling scheme (mean difference, amp/trial = −2.49 ± 0.29% GoF; *P* < 0.05, Tukey’s HSD test for multiple comparisons). Additionally, the two subtypes of mean amplitude weighted models did not differ from each other in terms of their GoF indices, and neither did the two subtypes of d-prime weighted models (*P* > 0.05, Tukey’s HSD tests for multiple comparisons; Figure 6B). Additionally, the maximal GoF achieved by the model also increased with time in all pooling schemes and plateaued at around 50–75 ms after stimulus onset (*n* = 1–512 neurons, 98–99% GoF; Figure 6C). Finally, the number of neurons needed to achieve the maximal GoF decreased with time in all pooling schemes, but only the uniform and the d-prime weighted pooling schemes were able to achieve maximal fitness (98–99% GoF) with fewer than 100 neurons (Figure 6D). Taken together, Figures 6A–D demonstrated that the d-prime weighted pooling strategies were the most optimal in terms of the overall performance, and this advantage over other pooling strategies was the most apparent in short integration time windows (25–50 ms).

### d-prime Weighted Pooling Scheme: Performance

**Figure 7:**
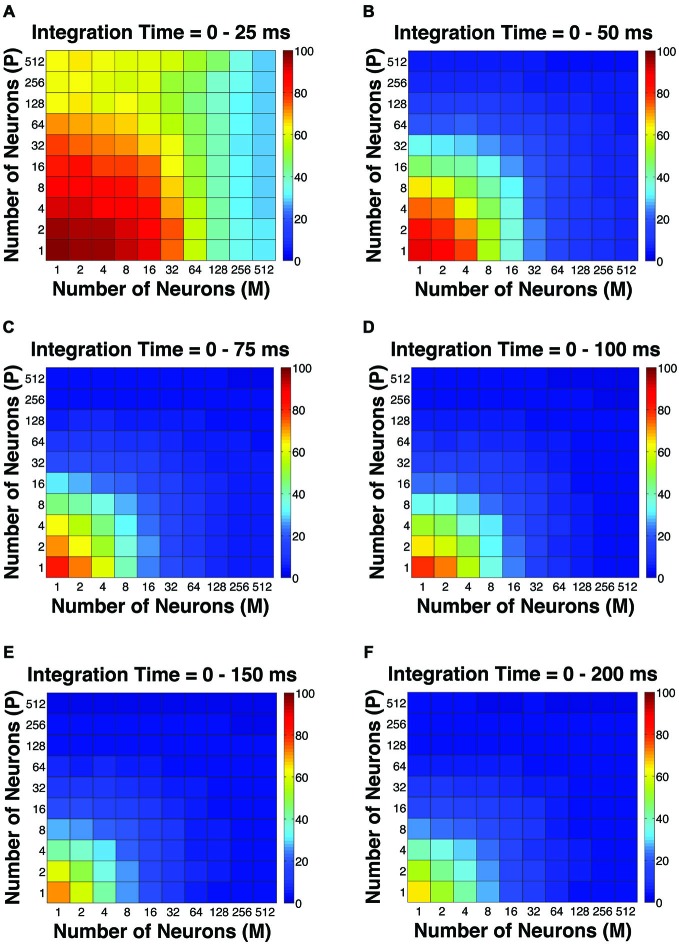
**The simulated “psychophysical” thresholds in the d-prime weighted scheme for different pool sizes (*n*) and integration time windows (*t*)**. Red indicates high threshold (100% contrast), whereas blue indicates low threshold (0% contrast). **(A)** *t* = 0–25 ms. **(B)** *t* = 0–50 ms. **(C)** *t* = 0–75 ms. **(D)** *t* = 0–100 ms. **(E)** *t* = 0–150 ms. **(F)** *t* = 0–200 ms.

Of all the alternative pooling strategies described above, we were the most interested in the d-prime weighted strategy because of its superior performance. In the next few sections we discuss in detail the performance, structure and properties of the d-prime model. First, in terms of the simulated psychophysical threshold, at extremely short integration time windows (25 ms), the d-prime model failed to achieve good psychophysical performance (i.e., threshold < 10% contrast) even when it incorporated a large number of neurons from both the P and M populations (*n* = 512 P neurons, 512 M neurons), but incorporating a large number of M neurons (*n* = 128–512) rather than P neurons was more beneficial to model performance (Figure 7A). At relatively brief intervals (50 ms), incorporating a large number of either P or M neurons (*n* = 256–512) could achieve good psychophysical performance (i.e., threshold < 10% contrast; Figure 7B). At medium to long intervals (75–200 ms), a wider range of M/P neuron combinations (*n* = 64–512) yielded good model performance (Figures 7C–F). Comparing Figure 7 (d-prime pooling) to Figure 2 (uniform pooling), it is clear that the d-prime model behaved rather similarly to the uniform pooling model in terms of its psychophysical performance, but there were apparent differences between the two models in the 25 ms and 50 ms time windows. Specifically, the d-prime model achieved much lower psychophysical thresholds than the uniform model in both time windows (25 ms: mean difference = −25.00 ± 1.13% contrast, *P* = 0.00, Wilcoxon signed rank test; 50 ms: mean difference = −4.25 ± 0.28% contrast, *P* = 0.00, Wilcoxon signed rank test).

**Figure 8:**
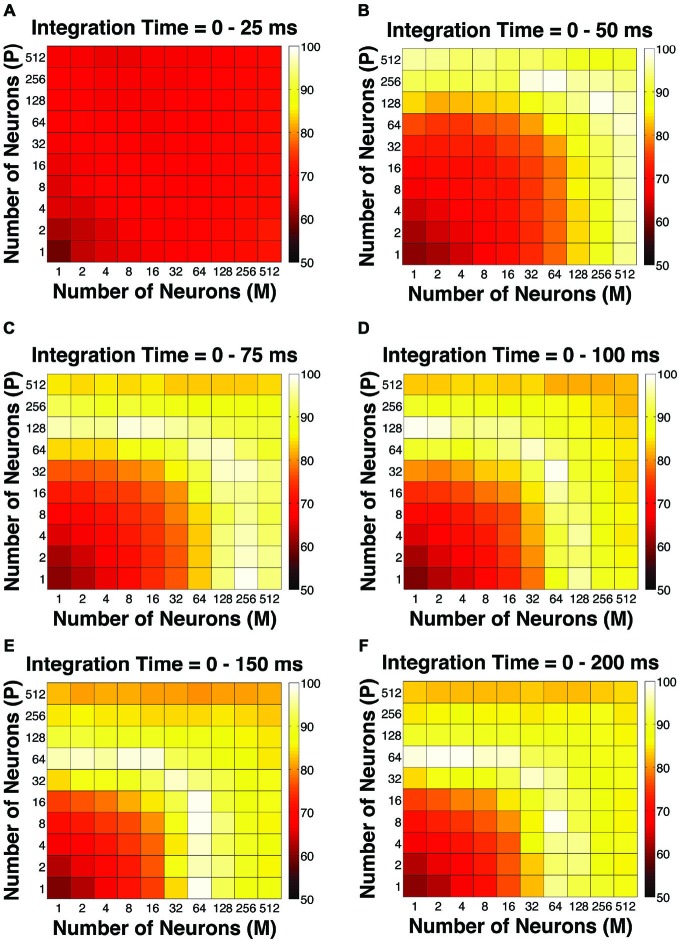
**The GoF indices in the d-prime weighted scheme for different pool sizes (*n*) and integration time windows (*t*)**. A GoF of 100% (white) indicates that the model perfectly matches the observed psychometric threshold as well as choice probabilities for both P and M neurons. **(A)** *t* = 0–25 ms. **(B)** *t* = 0–50 ms. **(C)** *t* = 0–75 ms. **(D)** *t* = 0–100 ms. **(E)** *t* = 0–150 ms. **(F)** *t* = 0–200 ms.

In terms of the overall fitness quantified as GoF, at extremely short integration time windows (25 ms), the d-prime model failed to reproduce the observed threshold and choice probabilities even when it incorporated a large number of neurons from both the P and M populations (*n* = 512 P neurons, 512 M neurons; Figure 8A). In 50 ms windows, incorporating a large number of either P or M neurons (*n* = 256–512) could explain the observed threshold and choice probabilities (Figure 8B). Finally, at medium to long intervals (75–200 ms), a wider range of M/P neuron combinations (*n* = 32–256) yielded good model performance, but further increasing the number of neurons would result in a decrease in model performance (Figures 8C–F). Comparing Figure 8 (d-prime pooling) with Figure 4 (uniform pooling), it is clear that the temporal evolution of the GoF index for the d-prime model resembled that for the uniform model, but there were apparent differences between the two models in the 50 ms and 75 ms time windows. To be more precise, in the 50 ms window, the d-prime model demonstrated better overall performance than the uniform model (mean difference = 4.85 ± 0.51% GoF, *P* = 0.00, Wilcoxon signed rank test). In the 75 ms window, in contrast, the overall performance did not differ between the two types of pooling models (mean difference = 0.00 ± 0.48% GoF, *P* = 0.30, Wilcoxon signed rank test), but the number of neurons needed to achieve good model performance (>90% GoF) was significantly reduced in the d-prime model (d-prime model: *n* = 64–256 neurons, uniform model: *n* = 128–512 neurons).

### d-prime Weighted Pooling Scheme: Structure

**Figure 9:**
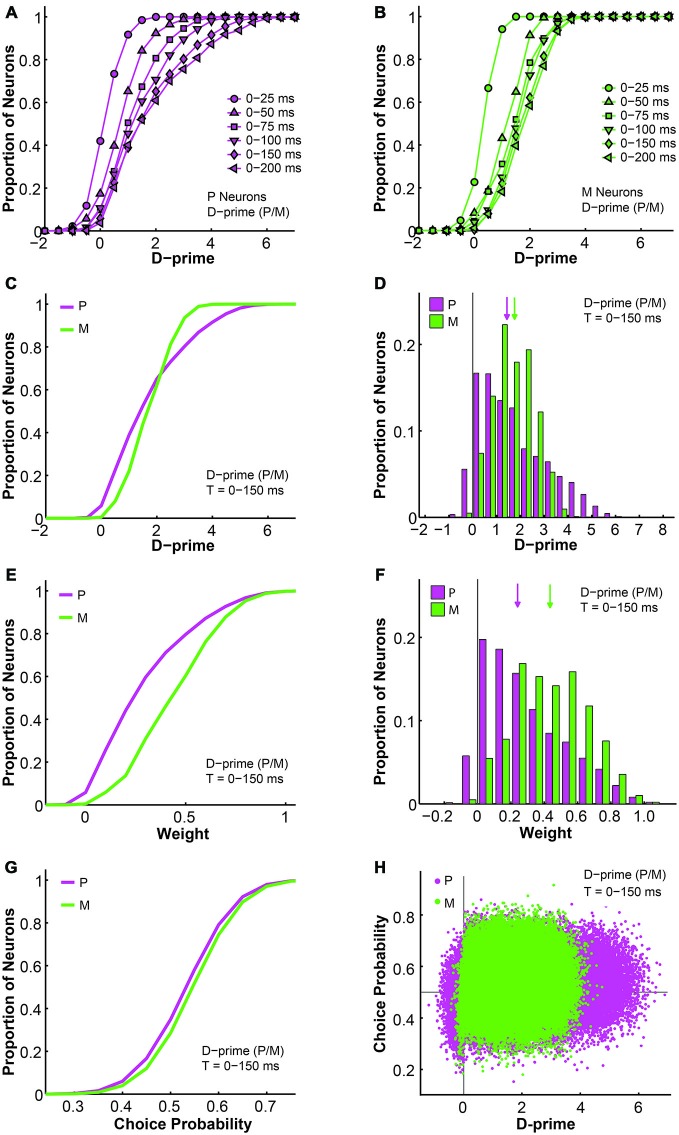
**Further analysis of the d-prime weighted pooling scheme: the relationship between d-prime, weight, and choice probability. (A,B)** Cumulative d-prime distributions for P (magenta, **A**) or M (green, **B**) neurons in different integration time windows (*n* = 512 neurons). **(C)** Cumulative d-prime distributions for P (magenta) and M (green) neurons in a 0–150 ms window (*n* = 512 neurons, 200 simulations). **(D)** D-prime distributions for P (magenta) and M (green) neurons in a 0–150 ms window (*n* = 512 neurons, 200 simulations). Arrow: median d-prime; solid line: d-prime = 0. **(E)** Cumulative weight distributions for P (magenta) and M (green) neurons in the same 0–150 ms window (*n* = 512 neurons, 200 simulations). **(F)** Weight distributions for P (magenta) and M (green) neurons in the same 0–150 ms window (*n* = 512 neurons, 200 simulations). Arrow: median weight; solid line: weight = 0.** (G)** Cumulative choice probability distributions for P (magenta) and M (green) neurons in the same 0–150 ms window (*n* = 512 neurons, 200 simulations). **(H)** Choice probability values were positively correlated with d-primes (*n* = 512 neurons, *t* = 0–150 ms, 200 simulations). Horizontal line: choice probability = 0.5; vertical line: d-prime = 0.

Next, we examined the relationship between d-primes, weights, and choice probabilities for different cell types within the d-prime pooling model. First, intuitively, as the model was allowed to integrate firing rate information for longer durations, the overall d-prime distributions extended accordingly, for both P and M populations (*F* = 87.74, *P* = 0.00, 2-way ANOVA main effect for time; Figures 9A,B). Next, in a fixed time window of medium duration (*n* = 512 neurons, *t* = 0–150 ms), we compared the d-prime distributions for P and M neurons. In P neurons, the average d-prime was 1.70 ± 0.00 and the median was 1.40. In M neurons, the average d-prime was 1.69 ± 0.00 and the median was 1.69 as well. Even though the average d-primes were similar, the shapes of the distributions differed dramatically between the two cell types, with the P d-prime distribution much more widely spread (P interquartile range = 2.05, M interquartile range = 1.25) and positively skewed (P skewness index = 0.73, M skewness index = 0.12; Figures 9C,D). In the same time window (*t* = 0–150 ms), the pooling weight of each individual neuron was directly determined by its d-prime value, and the weight distributions for P and M neurons were therefore very reminiscent of the their corresponding d-prime distributions in terms of shape. As the pooling weight of a neuron could not exceed 1.0, however, the weight distributions were scaled-down versions of the corresponding d-prime distributions (P weight: mean = 0.29 ± 0.00, median = 0.23; M weight: mean = 0.44 ± 0.00, median = 0.43). As a result, the weight distributions for P and M neurons still differed from each other in terms of skewness (P skewness index = 0.74, M skewness index = 0.15), but they were no longer distinguishable in terms of spread (P interquartile range = 0.35, M interquartile range = 0.32; Figures 9E,F).

We also analyzed the simulated choice probabilities for P and M neurons in the same time window (*t* = 0–150 ms), and found that the P and M choice probabilities in the d-prime model (P choice probability = 0.53 ± 0.00, M choice probability = 0.54 ± 0.00) resembled their experimentally measured counterparts (Jiang et al., 2015, also see above; Figure 9G). Furthermore, individual choice probability values were positively correlated with d-prime values for both P neurons (*r* = 0.08, *P* = 0.00) and M neurons (*r* = 0.04, *P* = 0.00; Figure 9H), indicating that the more sensitive LGN neurons were also more correlated with the behavioral choices of the monkeys.

### d-Prime Weighted Pooling Scheme: Which One to Choose?

As mentioned above, the d-prime weighted pooling scheme could be further divided into two subtypes depending on whether the P and M populations were weighted separately or together. These two types of d-prime models were indistinguishable from each other in terms of overall fitness, but we were interested in comparing their structures and detailed properties as well as making inferences as to which model was neurobiologically more meaningful. In the previous section we described the relationship between d-prime, weight, and choice probability in the scenario where P and M neurons were weighted separately according to their respective maximal d-primes, and in this section we perform similar analyses on the alternative d-prime model where P and M neurons were weighted together.

**Figure 10:**
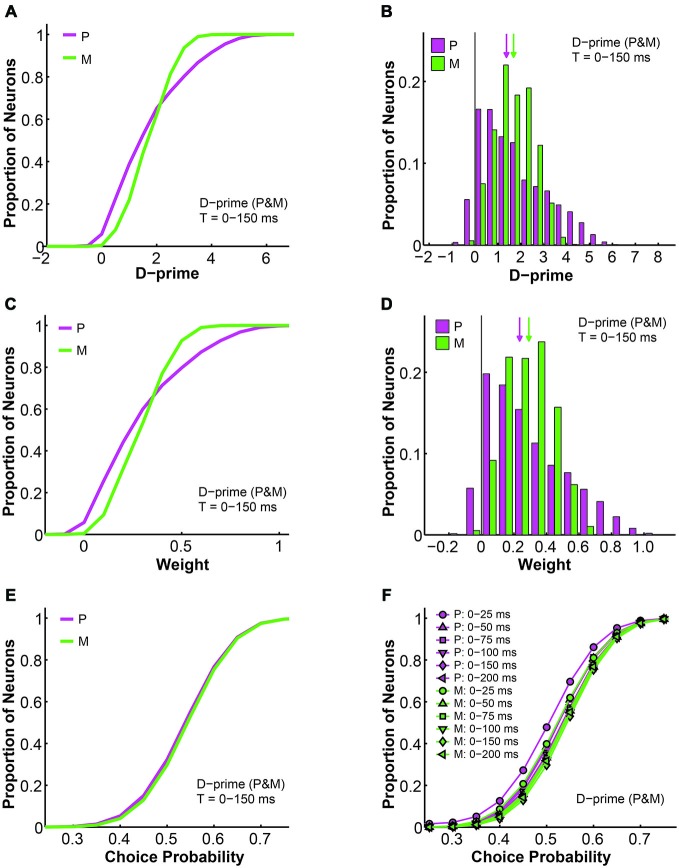
**Further analysis of the d-prime weighted pooling scheme: P and M neurons weighted together in reference to one maximal d-prime. (A)** Cumulative d-prime distributions for P (magenta) and M (green) neurons in a 0–150 ms window (*n* = 512 neurons, 200 simulations). **(B)** D-prime distributions for P (magenta) and M (green) neurons in a 0–150 ms window (*n* = 512 neurons, 200 simulations). Arrow: median d-prime; solid line: d-prime = 0.** (C)** Cumulative weight distributions for P (magenta) and M (green) neurons in the same 0–150 ms window (*n* = 512 neurons, 200 simulations). **(D)** Weight distributions for P (magenta) and M (green) neurons in the same 0–150 ms window (*n* = 512 neurons, 200 simulations). Arrow: median weight; solid line: weight = 0.** (E)** Cumulative choice probability distributions for P (magenta) and M (green) neurons in the same 0–150 ms window (*n* = 512 neurons, 200 simulations). **(F)** Cumulative choice probability distributions for P (magenta) and M (green) neurons in different integration time windows (*n* = 512 neurons).

First, as the d-prime value is a direct reflection of the signal-to-noise ratio of single neural responses, it is not surprising that the d-prime distributions remained the same regardless of the pooling strategy (compare Figures 9C,D to 10A,B). Specifically, for the P population, the average d-prime here was 1.70 ± 0.00 and the median was 1.40. For the M population, the average d-prime here was 1.70 ± 0.00 and the median was 1.70 as well. Additionally, the P and M d-prime distributions differed significantly in their shapes, with the P d-prime distribution much more widely spread (P interquartile range = 2.04, M interquartile range = 1.25) and positively skewed (P skewness index = 0.73, M skewness index = 0.11; Figures 10A,B). The weight distributions for P and M neurons in the same time window (*t* = 0–150 ms), however, were very different between the two types of d-prime models (compare Figures 9E,F to 10C,D). Specifically, when P and M neurons were pooled together, as was the case here, the weight distributions were still scaled-down versions of their corresponding d-prime distributions (P weight: mean = 0.29 ± 0.00, median = 0.24; M weight: mean = 0.29 ± 0.00, median = 0.29), but both distributions retained their shape and spread. In other words, the weight distributions for P and M neurons still differed from each other in terms of both spread (P interquartile range = 0.36, M interquartile range = 0.21) and skewness (P skewness index = 0.74, M skewness index = 0.14; Figures 10C,D). Two-way ANOVAs confirmed that while the d-prime distributions did not differ (*F* = 0.01, *P* = 0.91, 2-way ANOVA main effect for pooling strategy), the weight distributions differed dramatically between the two pooling schemes (*F* = 12073.18, *P* = 0.00, 2-way ANOVA main effect for pooling strategy). This difference in the weight distributions was presumably due to the fact that, compared with M d-prime distributions, P d-prime distributions were more widely spread with greater maximal values. Thus, when P and M populations were scaled together, as was the case here, both were most likely scaled in reference to the d-primes of a subset of P neurons, thus preserving the shapes as well as spreads of these distributions. When P and M populations were scaled separately, as was the case above, M neurons were scaled to a lesser degree when compared with P neurons, rendering the spreads of the two distributions indistinguishable.

We also analyzed the simulated choice probabilities for P and M neurons in the same time window (*t* = 0–150 ms), and found that in this d-prime model the P and M choice probabilities (P choice probability = 0.53 ± 0.00, M choice probability = 0.54 ± 0.00) also resembled their experimentally measured counterparts (Jiang et al., 2015, also see above; Figure 10E). Furthermore, these P and M choice probability distributions developed throughout the 200 ms stimulus presentation time (*n* = 512 neurons, *F* = 3.78, *P* = 0.00, 2-way ANOVA main effect for time), much like in the other d-prime model (compare Figures 5H to 10F), and confirming the temporal dynamics that we had observed in the LGN of awake monkeys (Jiang et al., 2015).

## Discussion

We previously reported that, in a 2AFC contrast detection task, single LGN P and M neurons demonstrated significant choice probabilities despite their relatively poor neural sensitivities (Jiang et al., 2015). In this study, we examined quantitatively the effects of the neural pool size, the Fano factor, the interneuronal correlation and the downstream pooling noise on the simulated psychophysical performance and choice probability values. We investigated different pooling/readout schemes that ranged from basic, uniform pools to more optimal pools that preferably weighted the more sensitive single neurons. We compared these pooling strategies in integration time windows of different durations, and found that the most successful model consisted of a medium number of LGN neurons (n = ~30–250) in medium to long integration time windows (75–200 ms), with individual neurons weighted differentially according to their d-prime values. These results indicated that both the psychophysical threshold and the LGN choice probabilities during contrast detection could be fully explained using simple, bottom-up pooling models without assuming significant interneuronal correlations, and that such modeling efforts helped elucidate the complicated relationship between neural sensitivity, readout weight, and choice probability. We now consider the significance of these results in light of previous experimental and theoretical findings.

### Pooling/Readout Strategies

The primate LGN provides major feedforward input to the visual cortex, and is an essential thalamic gateway to conscious vision (Sherman and Guillery, 2001; Jones, 2007; Schmid et al., 2010; Casagrande and Ichida, 2011; Saalmann and Kastner, 2011). The effort to understand how the information carried by LGN cells is utilized at later stages is therefore of great importance. Very generally, pooling /readout rules can be divided into two categories. In the first, perceptual decisions are based on signals provided by one or several of the most sensitive sensory neurons (i.e., lower envelope principle; Barlow, 1995). In the second category, perceptual decisions are based on some form of pooled responses from many sensory neurons. The uniform pooling as well as alternative weighted pooling schemes used in this paper all fall into the second category.

The lower envelope principle, however, always remains a theoretical possibility. This is because even in a detection task such as ours, where the sensitivities of most single neurons failed to match the psychophysical sensitivity of the subject, there were still a small but significant proportion (13.5%; Jiang et al., 2015) of single cells that matched or even outperformed the subject. That being said, if the lower envelop principle were true, we would expect a choice probability distribution that is qualitatively different from what was observed in physiological recordings. Briefly, if only a few neurons contribute to a perceptual decision, all of them should demonstrate very significant choice probabilities, with the rest of the entire neural population exhibiting chance choice probabilities (Nienborg et al., 2012; Haefner et al., 2013; but see below for the influence of interneuronal correlation on choice probability). In reality, most cortical recordings have reported a broad distribution of weakly significant choice probabilities (for example, see Britten et al., 1996; Uka and Deangelis, 2004; Liu and Newsome, 2005; Purushothaman and Bradley, 2005; Nienborg and Cumming, 2006; Palmer et al., 2007; Price and Born, 2010; Liu et al., 2013), a result that was confirmed in the LGN (Jiang et al., 2015).

Consequently, our current study as well as a number of other computational studies (Shadlen et al., 1996; Purushothaman and Bradley, 2005; Cohen and Newsome, 2009; Haefner et al., 2013) arrived at the conclusion that an ideal perceptual decision pool consists of not just a few, but rather tens to hundreds of single sensory neurons. In this type of broad decision pool, the readout weight profile, or pooling strategy, of the neural system can be inferred from experimentally measurable quantities such as the behavioral threshold and the choice probability distributions (Haefner et al., 2013; Liu et al., 2013), as demonstrated in the current study.

### The d-Prime Weighted Pooling Model

According to signal detection theory (Green and Swets, 1966), d-prime is one of the most useful and widely used descriptors of signal-to-noise ratio. The d-prime model was one of several selective weighted pooling models that we examined in this paper. In this model the readout weight of each neuron was determined by its d-prime value at high contrast, with the neuron with the greatest d-prime value carrying a weight of 1.0. Our simulations showed that the d-prime weighted model provided a parsimonious and complete account of all of our experimental data including the monkeys’ psychophysical performance and the population distributions of LGN choice probabilities.

Compared with the simple uniform pooling scheme, the d-prime model was superior in several major ways: (1) The d-prime model achieved lower average and minimal psychophysical thresholds (Figures 5A–C), especially in shorter integration time windows; (2) The d-prime model more faithfully reflected the temporal developments of choice probabilities in LGN P and M neurons (Figure 5H); and (3) The d-prime model achieved greater average and maximal model fitness (Figures 6A–C) with fewer neurons (Figure 6D), especially in shorter time windows. Additionally, the d-prime weighted model also demonstrated a clear, direct relationship between choice probability and neural sensitivity (Figure 9H), indicating that neurons with higher signal-to-noise ratios were also more correlated with perceptual choices. This correlation was even more pronounced in shorter integration time windows, where fewer neurons demonstrated high signal-to-noise ratios (e.g., d-prime vs. choice probability, *t* = 0–25 ms: r for P neurons = 0.22, *P* = 0.00; r for M neurons = 0.28, *P* = 0.00. *t* = 0–50 ms: r for P neurons = 0.18, *P* = 0.00; r for M neurons = 0.18, *P* = 0.00). According to previous theoretical work (Haefner et al., 2013; Moreno-Bote et al., 2014), when choice probabilities and neural sensitivities (i.e., d-primes) exhibit such direct correlations, it is an indication that the pooling/readout strategy is optimal for the task. Last but not least, the uniform pooling scheme assumes that even after extensive practice of a perceptual task, the initial pattern of widespread and diffuse synaptic connections will remain unrefined. In reality, however, perceptual learning is known to dramatically alter the properties of single sensory neurons (Sasaki et al., 2010; Kumano and Uka, 2013; Watanabe and Sasaki, 2015). Therefore, neurobiologically speaking, the d-prime weighted model is also the more plausible solution *in vivo*.

If the d-prime weighted pooling strategy is indeed utilized in the neural system, our simulations make several specific predictions that can be tested in future psychophysical and physiological recordings: (1) A single LGN neuron’s d-prime value should be directly correlated with its choice probability (Figure 9H), and this correlation should be stronger in shorter integration time windows (see above); (2) A single LGN neuron’s d-prime and choice probability values should both develop throughout the stimulus presentation time (see Figures 5H, 9A,B, 10F). This prediction was already confirmed in our previous publication (Figures 7C,D in Jiang et al., 2015); and (3) More importantly, if LGN responses are optimally pooled in subsequent stages, humans and monkeys should be able to maintain the same contrast detection performance with stimulus durations as short as 50–75 ms (see Figures 5A–C), even though LGN choice probabilities may decrease in such short integration time windows (Figures 5H, 10F).

As mentioned above, the d-prime weighted pooling model could be further divided into two types, with one weighing P and M neurons separately according to their respective maximal d-primes, and one weighing P and M neurons together according to one maximal d-prime. These two models were indistinguishable in terms of their overall performance, but they did differ from each other in their readout weight and choice probability distributions. When considered separately, M neurons were significantly more heavily weighted than P neurons (Figure 9F). In contrast, when weighted together, M neurons only had a very slight advantage over P neurons (Figure 10D). Computationally, we could not rule out one model in favor of the other. Neurobiologically, the former scenario is more likely to occur only in layer 4 of V1, where LGN P and M inputs remain segregated (Casagrande and Xu, 2004; Nassi and Callaway, 2009). The latter readout scheme, in contrast, is more likely to occur everywhere else in the cortex, where LGN P and M inputs are mixed and integrated.

### The Limitations of the Pooling Models

First, to appropriately interpret our modeling results, it is important to understand the role of interneuronal correlation in perceptual decision making. In medium-sized decision pools such as ours, interneuronal noise correlations can strongly influence not only the choice probability structure, but also the readout weight distribution (Chen et al., 2006; Haefner et al., 2013). In cortex, interneuronal correlations are considered to be mostly unavoidable (Averbeck et al., 2006; Cohen and Kohn, 2011) because of the extensively shared connections between neurons (Zohary et al., 1994; Shadlen and Newsome, 1998; Bair et al., 2001; Reich et al., 2001; Averbeck et al., 2006; Cohen and Maunsell, 2009). Recent studies, however, reported overall interneuronal correlations not different from chance in chronic recordings from large populations of V1 neurons in awake monkeys (Ecker et al., 2010, 2014). Compared to the visual cortex, neural circuitry in the LGN is simpler (Casagrande and Norton, 1991; Nassi and Callaway, 2009) and highly specific to cell types (Casagrande and Xu, 2004; Briggs and Usrey, 2011; Ichida et al., 2014). Furthermore, cognitive factors such as attention (Cohen and Maunsell, 2009; Mitchell et al., 2009) and perceptual learning (Gu et al., 2011) are known to decrease the existing interneuronal correlations in a perceptual decision task. It is therefore not entirely surprising that we found LGN interneuronal correlations to be not significantly different from 0.0 during a contrast detection task (Jiang et al., 2015).

Even though we were able to successfully model experimentally measured psychophysical performance and choice probabilities without assuming any significant interneuronal correlations, we could not rule out the possibility that, in reality, there exist some fine patterns within the LGN interneuronal correlation structure. In fact, recent modeling work has revealed that it is not the average interneuronal correlation level, but the structure of a specific type of differential correlation, that determines choice probability values in a perceptual decision pool (Haefner et al., 2013; Moreno-Bote et al., 2014). Briefly, in cortex, interneuronal correlations are known to be stronger for similarly tuned neurons rather than dissimilarly tuned ones (Zohary et al., 1994; Maynard et al., 1999; Averbeck and Lee, 2003; Gu et al., 2011; Adibi et al., 2013). In this scenario, the neurons at the center of the decision pool could have the largest choice probabilities simply because they are most correlated with all the other neurons in the same pool. In other words, choice probabilities could decrease in the direction of the pool boundaries solely because of the correlation structure, but not the readout weight structure, of the decision pool (Chen et al., 2006; Haefner et al., 2013). This is a possibility that we did not model, and therefore could not rule out for the LGN perceptual decision pool. Furthermore, thalamic interneuronal correlations may be qualitatively different from those measured in the cortex, as LGN neurons sharing the same retinal inputs are known to exhibit very strong temporal correlations in their firing patterns (Alonso et al., 1996; Dan et al., 1998). This is also a possible correlation structure that we did not explore in our models, and as a result we could not rule out its potential influence on the psychophysical sensitivity and choice probabilities of LGN neurons.

Additionally, our pooling models were abstract representations of the minimal computations required to account for our experimental data. These models did not specify and were not critically dependent on, for example, exactly when and where a perceptual “choice” is made *in vivo*. Furthermore, even though we were able to simulate neural pools of infinitely large sizes for a large number of trials, the accuracies of these simulations were constrained by the sample sizes in our original experimental data (Jiang et al., 2015). Finally, although we took into consideration the temporal evolution of a variety of critical factors such as the mean and variance of neural response, the d-prime, and the choice probability, we did not characterize how temporal changes in other parameters such as the interneuronal correlation and the downstream pooling noise might influence model performance. Despite these limitations, we believe that our modeling results clearly and unarguably support the hypothesis that the neural pool consists of not just a few very sensitive neurons but many neurons, likely 100 or more, at the level of the visual thalamus, and that the response fluctuations in these thalamic neurons can influence perception, with the more sensitive neurons exerting a bigger influence on perception.

## Author Contributions

YJ, GP and VC conceptualized and designed the study, YJ collected and analyzed the data, YJ and VC interpreted the results, YJ drafted the manuscript, YJ and VC revised the manuscript, YJ, GP and VC approved the final version of the manuscript and agreed to be accountable for all aspects of the work.

## Funding

This work was supported by National Institutes of Health from grants EY001778 (VAC), EY025422 (VAC), R21 EY019132 (VAC), core grants EY008126 and HD15052, and funds from the Department of Cell and Developmental Biology at Vanderbilt University.

## Conflict of Interest Statement

The authors declare that the research was conducted in the absence of any commercial or financial relationships that could be construed as a potential conflict of interest.
